# Size Effect and Material Property Effect of the Impactor on the Damage Modes of the Single-Layer Kiewitt-8 Reticulated Dome

**DOI:** 10.1155/2013/848347

**Published:** 2013-08-20

**Authors:** Li Lin, Feng Fan, XuDong Zhi, HongFeng Yin

**Affiliations:** ^1^School of Civil Engineering, Harbin Institute of Technology, No. 77 Huanghe Road, Nangang District, Harbin 150090, China; ^2^College of Civil Engineering and Architecture, Harbin University of Science and Technology, No. 52 Xuefu Road, Nangang District, Harbin 150080, China

## Abstract

The dynamic response of large space structures under accidental impact has been the subject of intense research since the occurrence of the 9/11 incident. In the present paper, using the 3D ANSYS/LS-DYNA, size effect and material property effect of the impactor on the damage modes of the single-layer Kiewitt-8 reticulated dome were investigated, respectively, where the impactor was the cylinder and the impact direction was vertical. Firstly, analytical results with the rigid impactor indicated that the impactor size can change the damage mode of the reticulated dome. It was found that the probability happening to the global collapse has an obvious rise with the size increase of the impactor. Furthermore, the deformable impactor was considered to figure out the difference with the rigid impactor; the comparisons indicated that the deformable impactor, which has the same mass and the same striking velocity with the rigid impactor, can contribute to the occurrence of the global collapse at a certain initial striking condition.

## 1. Introduction

 Shock dynamics mechanics of large space structure has gained researchers' attention since the occurrence of the 9/11 incident. Large space structures are usually such symbolic buildings of social and economic importance. It is very necessary and beneficial to investigate the dynamic behaviour of large space structures, aiming to look for the strategy to avoid the global collapse. Yet there are little study work approaching to the dynamic behaviour of this kind of structures under impact.

Under accidental impact, lots of previous studies and investigations concentrated mainly on the frames and high-rise buildings; typical researches could be found in Bodner and Symonds [1], Zhou et al. [2], Samuel [3], and Lynn and Isobe [4]. Yet, the work on the large space structures is relatively few. Analysis of single-layer Kiewitt-8 reticulated domes with low speed and small mass impact was conducted [5–7]. Recently, Fan and his colleagues performed a series of studies investigating behaviour of single-layer Kiewitt-8 reticulated domes under the impact theoretically and numerically [8, 9]. 

It is noted that the previous studies on single-layer Kiewitt-8 reticulated domes were paid few attention to the impact of small size, and it is assumed mostly that the impactor was rigid. Thus, in this work, size effect of the impactor to the dynamic response of the reticulated dome was conduct based on 3D numerical simulations. Meanwhile, the differences between the rigid impactor and the deformable impactor were analysed. 

## 2. Numerical Models

**Figure 1:**
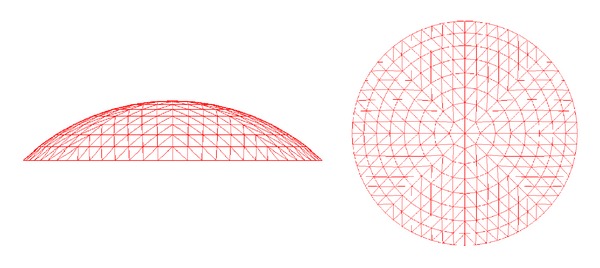
Simulation model of the single-layer Kiewitt-8 reticulated dome.

**Figure 2:**
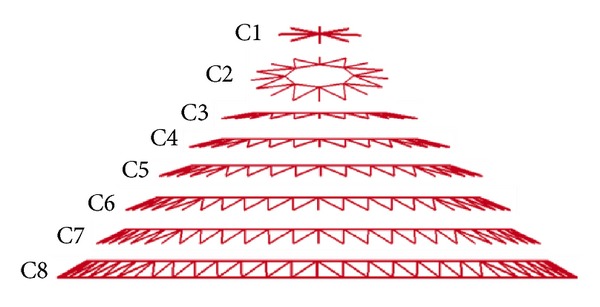
Labeled number of every segment on the reticulated dome.

All the simulations in the study were performed with LSDYNA-3D code.  Figure 1 shows a single-layered Kiewitt-8 reticulated dome with a 60 m span. The dome consists of eight latitudinal circles and eight main radial ribs that divide the sphere into 8 axisymmetrical fan-shaped segments, which are labeled to make the numerical results more clearly in Figure 2. Diagonal members are applied to link the latitudinal and the main radial members, and similar triangular grids are thus formed all over the spherical surface. In the present work, two kinds of roof load were involved, as given in Table 1.

For the rigid impactor, in order to assure that the impactor does not deform plastically during the impact process, its strength was artificially set to a relatively high value of 20 GPa in all simulations. 

Reversely, for the deformable impactor, the material property is the same for the dome structure. Since impact is related to the high strain rate, which is a very important role to the dynamic behaviour of structures, thus the constitute model of piecewise JC was adopted for the dome structure and the deformable impactor, which can take into account the strain-rate effect to some degree. The initial yield stress was 207 MPa, the elastic modulus was 206 GPa, the Poisson's ratio was 0.3, and the effective plastic strain at failure was defined as 0.25 [10].

In the following section, the impact events with the rigid impactor are named as the rigid impactor; similarly, the impact events with the rigid impactor took the place of the deformable impact.

## 3. Analysis of Damage Modes

Herein assumptions for damage analysis needs to be given: (1) heat energy and gravity are not taken into account; (2) the friction between the impactor and the curved beam is neglected.

**Figure 3:**
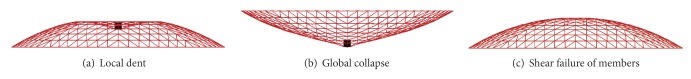
Damage modes of single-layer Kiewitt-8 reticulated dome.

Based on the model developed above, the damage characteristic of the dome with the initial kinetic energy was investigated. The numerical simulation results from the rigid impact and the deformable impact both indicated that these damage modes could be concluded according to the dynamic responses, as shown in Figure 3.

Mode 1: local dent. Due to the low initial kinetic energy, the impactor does not perforate the reticulated dome, and the members on top of the reticulate dome are damaged locally without the break occurrence among the beams. The affect zone on the top of the reticulated dome is small in comparison with the whole structure. The final deformation is shown in Figure 3(a). 

Mode 2: global collapse. This damage is global, which can cause the whole structure collapse. Under this impacting load, the top of the reticulated dome moves down until touching the ground, the members around the top members are pull down, as described in Figure 3(b). On this damage mode, there exist two scenarios: global collapse without the plug and global collapse with the plug. The plug is due to the fact that the members around the impactor happen to break, when the impactor reaches a certain height, as shown in Figure 6.

Mode 3: shear failure of members. Different from Mode 2, there is only the punching plug without the downwarp phenomenon in this damage mode. The members hit directly by the impactor are broken instantly when the initial kinetic energy is large enough. For the instantaneous damage, the affected zone of the impact load is small in comparison with the other damage modes. The typical damage form is given in Figure 3(c).

## 4. Size Effect of the Impactor to the Damage Modes

For the purpose of examining the effect of the impactor size, three kinds of different size impactors were chosen. The impactors with the diameters 2 m, 5 m, and 10 m were applied to the impact, where the cylinder height with 2 m was kept the same. By changing the density of the impactor, a series of different impact events with keeping the same striking kinetic energy were carried out. The range of the impactor mass and the striking velocity were, respectively, from 1 T to 50 T and from 5 m/s to 400 m/s.

Herein, it is necessary to point out that the impactor was assumed to be rigid, aiming to exclude the material property effect of the impactor.

**Figure 4:**
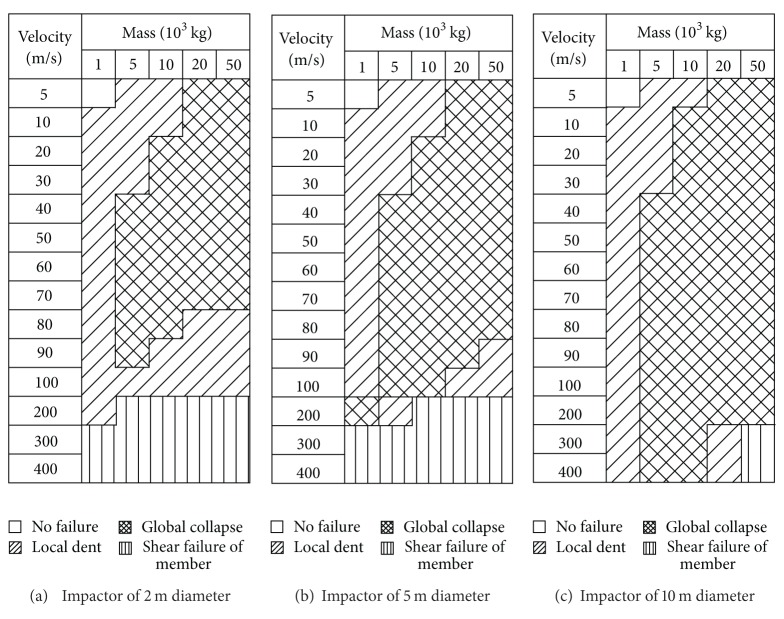
Damage modes of the impact events with the roof load of 120 kg/m².

**Figure 5:**
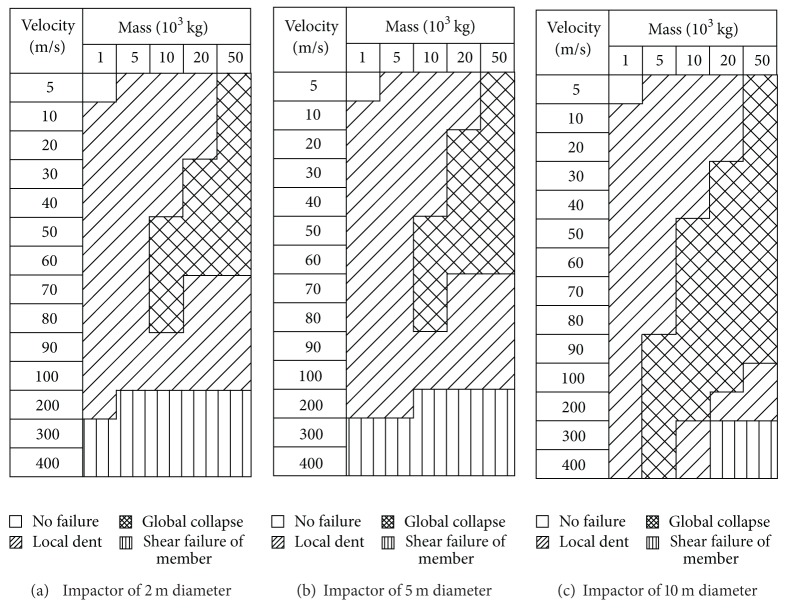
Damage modes of the impact events with the roof load of 60 kg/m².

The variations of damage modes with the mass and striking velocity of the impactor are given in Figures 4 and 5. It was found that the impactor size plays a key role in controlling the damage mode of the reticulated dome structure concerned in the present paper. As shown in Figures 4 and 5, the scope of Mode 2 gradually becomes larger with the increase of the impactor size. Conversely, the scope of Mode 3 has a descended trend with the increase of the impactor size. On the other hand, by comparisons between Figures 4 and 5, one deduces clearly that the load of roof can enhance the probability happening to the Mode 2.

**Figure 6:**
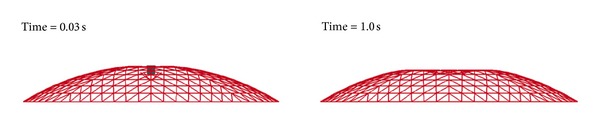
Damage process of the impact event with impactor of 2 m diameter and 10 T mass and the striking velocity of 100 m/s.

**Figure 7:**
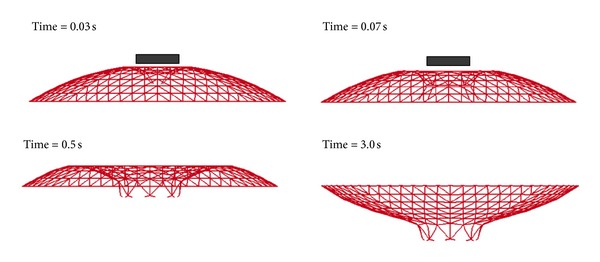
Damage process of the impact event with impactor of 10 m diameter and 10 T mass and the striking velocity of 100 m/s.

Aiming to make the analysis more clearly, two typical impact events with the same striking velocity and the same mass of the impactor were discussed in detail, where impactors with 2 m diameter and 10 m diameter were compared. The mass and the striking velocity of the impactor were, respectively, 10 T and 100 m/s. The damage processes are shown in Figures 6 and 7. From the damage features, it could be concluded that the impact event with impactor of 2 m diameter belongs to the Mode 3. Matching with the characteristic of Mode 2, the impact event with impactor of 10 m is in the range of Mode 3. Thus, it is indicated that the change of the impactor size with the same initial striking kinetic energy can lead to the variation of the damage mode.

**Figure 8:**
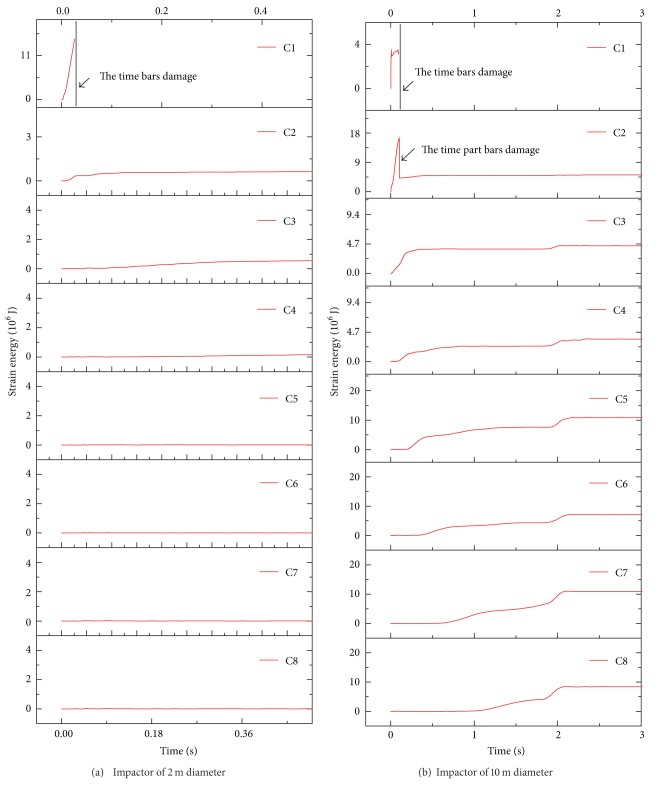
Time history of strain energy on two typical impact events with two different impactors and the same initial striking kinetic energy.

Furthermore, in terms of the strain energy, it was found that for the impact event with the impactor of 2 m diameter, the strain energy of the C1 has a dramatic increase, the strain energy of the C2 and C3 exhibits a smooth time history, and the value of the strain energy is relatively small. It seems to be zero on the strain energy of the other parts from Figure 8. Thus, it is demonstrated that the damage is concentrated on the C1. On the other hand, Figure 8 shows that for the impact event with the impactor of 10 m diameter, every segment of this structure happens to the deformation in light of the observation of the strain energy, and the strain energy of the C2 has a dramatic increase and the peak is relatively large; this leads us to get the conclusion that the members of the C2 are broken during the impact process. This furtherly indicates that the deformation of the impact event with the impactor of 10 m diameter is global. Finally, comparisons of the strain energy on two typical impact events prove again that the impactor size has an important effect on the damage mode of the reticulated dome.

## 5. Property Effect of the Impactor

In Section 4, size effect of the impactor was analysed, where the impactor was assumed to be rigid. The material property of the impactor was ignored. But in reality the deformable impactor was common; it is necessary to investigate the dynamic response of the dome structure subjected to the deformable impact.

Thus, in this section, the work on the deformable impact was approached. The material property is kept the same with that of the dome structure. The mass of the impactor is changed by adjusting the geometry parameters. The detailed information on the deformable impactor is given in Table 2.

**Figure 9:**
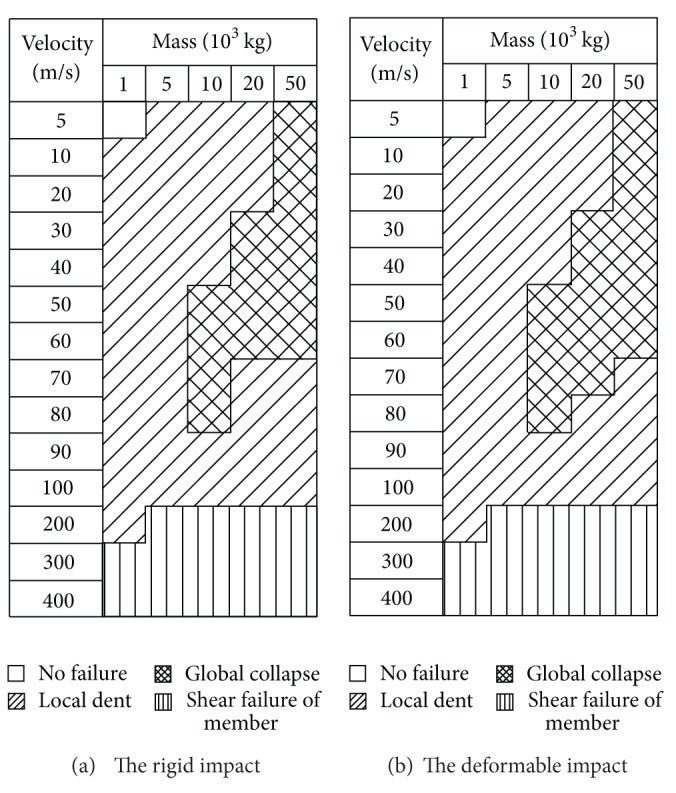
Comparison of the impact events with the roof load of 60 kg/m² between the rigid impact and the deformable impact.

**Figure 10:**
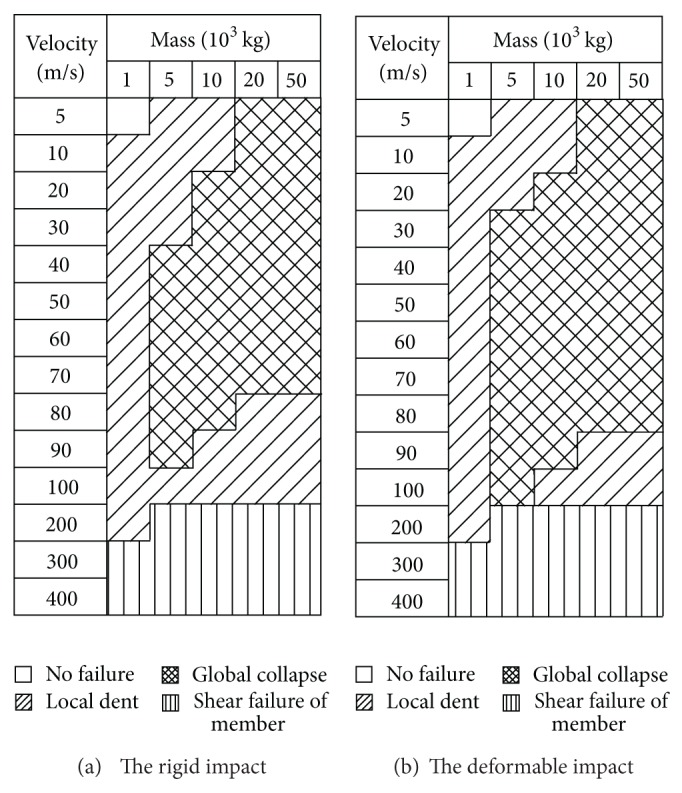
Comparison of the impact events with the roof load of 120 kg/m² between the rigid impact and the deformable impact.

As described in Section 3, the damage modes of the dome structure against to the deformable impact are similar to those of the rigid impact. The final analytical results are given in Figures 9 and 10. It is found that for the dome structure with the roof load of 60 kg/m², there is no difference between the rigid impact and the deformable impact. But, when the roof load is 120 kg/m², the distribution of the damage modes happens to the change: the area of the global damage mode becomes larger than that of the rigid impact. 

Aiming to check the reason resulting in the difference, two typical cases were discussed, where the roof load was 120 kg/m², and the mass and the striking velocity are 10 T and 90 m/s, respectively; the corresponding geometry parameters can be gained in Table 2. 

**Figure 11:**
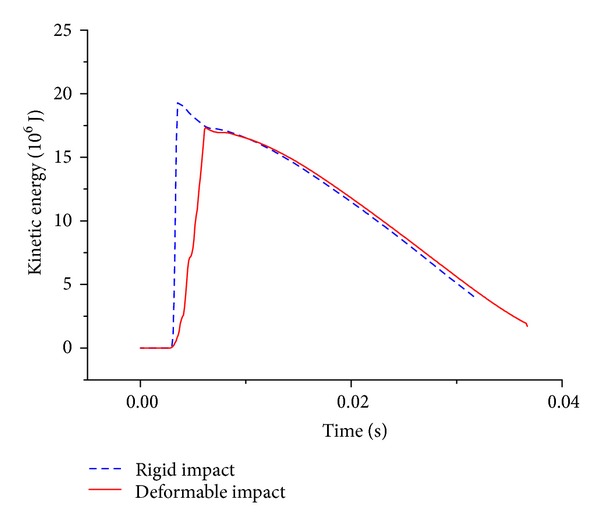
Kinetic energy of C1 segment.

**Figure 12:**
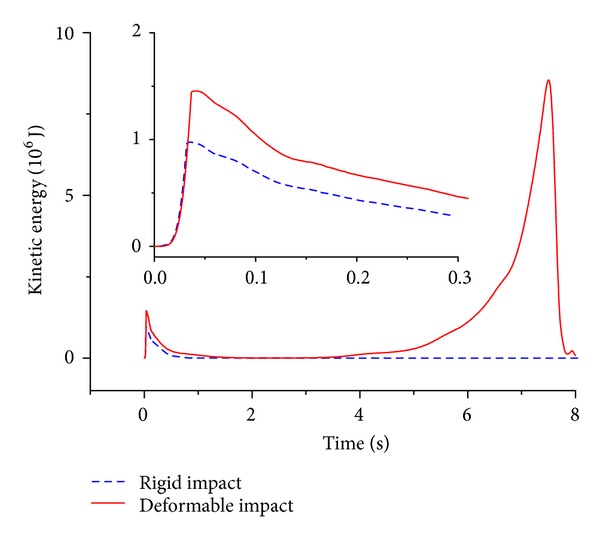
Kinetic energy of the unimpacting region.

Figure 9 shows that at this kind of initial striking condition the case with the rigid impact is corresponding with the local damage mode; reversely, the case with the deformable impact is characteristic of the global damage mode. It is found that the variation of C1 strain energy on the case with the deformable impact exhibits a milder trend than that of the case with the rigid impact, as described in Figure 11. And total strain energy of other segments emerges two peaks for the case with the deformable impact in Figure 12; this indicates that the energy gains the deliver from C2 segment to C8 segment. Thus it could be summarized that the difference between the rigid impact and the deformable impact mainly attributes to the succession of the energy from the structure top to the bottom. 

## 6. Conclusions

In the present paper, a series of 3D numerical simulations were conducted to investigate the dynamic response of the single-layer Kiewitt-8 reticulated dome against the rigid impact and the deformable impact, where the rigid impact means that the impactor is set to be rigid; reversely, the deformable impact is corresponding to the impact event that the impactor is deformable during the impact process. Firstly, based on the deformation features from the rigid impact and the deformable impact, three damage modes were identified, which are local dent, global collapse, and shear failure of members, respectively. Furtherly, based on the simulation results from the rigid impact, comparisons of damage modes among impact events indicate that the probability happening to the global collapse has a rise trend with the increase of the impactor size. And this conclusion gets the further validation by the analysis of the strain energy of the typical impact events during the impact process. 

On the other hand, the difference between the rigid impact and the deformable impact was conducted, and it was found that for the dome structure with the roof load of 120 kg/m², the deformable impact more easily contributes to the occurrence of the global damage mode, and the motivation driving to the difference is mainly from the energy deliver from the structure top to the bottom.

## Figures and Tables

**Table 1:** Geometry parameters of single-layered kiewitt-8 reticulated dome.

Latitudinal and radial member (mm)	Diagonal member (mm)	Span (mm)	Rise-to-span ratio	Roof load (kg/m^2^)
Φ180 × 7.0 mm	Φ168 × 6.0 mm	60	1/7	60/120

**Table 2:** Deformable impactors analysed.

Mass (T)	1	5	10	20	50
Diameter (m)	0.543	0.928	1.170	1.474	2.000
Height (m)	0.552	0.943	1.189	1.498	2.033
